# Portable and Battery-Powered PCR Device for DNA Amplification and Fluorescence Detection

**DOI:** 10.3390/s20092627

**Published:** 2020-05-05

**Authors:** Junyao Jie, Shiming Hu, Wenwen Liu, Qingquan Wei, Yizheng Huang, Xinxin Yuan, Lufeng Ren, Manqing Tan, Yude Yu

**Affiliations:** 1 State Key Laboratory of Integrated Optoelectronics, Institute of Semiconductors, Chinese Academy of Sciences, P.O. Box 912, Beijing 100083, China; jiejunyao@semi.ac.cn (J.J.); hushiming@semi.ac.cn (S.H.); liuwenwen@semi.ac.cn (W.L.); qingquanwei@semi.ac.cn (Q.W.); yizhuang@semi.ac.cn (Y.H.); yuanxinxin@semi.ac.cn (X.Y.); renlf@semi.ac.cn (L.R.); mqtan@semi.ac.cn (M.T.); 2 College of Materials Science and Opto-Electronic Technology, University of Chinese Academy of Sciences, Beijing 100049, China

**Keywords:** portable PCR device, thin-film heater, RTDs, Li-ion battery-powered

## Abstract

Polymerase chain reaction (PCR) is a technique for nucleic acid amplification, which has been widely used in molecular biology. Owing to the limitations such as large size, high power consumption, and complicated operation, PCR is only used in hospitals or research institutions. To meet the requirements of portable applications, we developed a fast, battery-powered, portable device for PCR amplification and end-point detection. The device consisted of a PCR thermal control system, PCR reaction chip, and fluorescence detection system. The PCR thermal control system was formed by a thermal control chip and external drive circuits. Thin-film heaters and resistance temperature detectors (RTDs) were fabricated on the thermal control chip and were regulated with external drive circuits. The average heating rate was 32 °C/s and the average cooling rate was 7.5 °C/s. The disposable reaction chips were fabricated using a silicon substrate, silicone rubber, and quartz plate. The fluorescence detection system consisted a complementary metal-oxide-semiconductor (CMOS) camera, an LED, and mirror units. The device was driven by a 24 V Li-ion battery. We amplified *HPV16E6* genomic DNA using our device and achieved satisfactory results.

## 1. Introduction

Polymerase chain reaction (PCR) is a powerful technique to amplify the specific DNA fragments in vitro, which can produce a large amount of target DNA from a very small amount of template DNA in 2 h [1,2,3]. PCR has been universally used in hospitals and research institutes for molecular biology study and is widely used in several fields such as gene analysis [4,5], clinical diagnoses [6,7], food safety [8,9], and forensic identification [10,11]. In the last few decades, human health and public health have received extensive attention, and there is an urgent need for pathogenic bacteria diagnoses to prevent massive contagion outbreaks in high-prevalence areas of infectious diseases such as gathering places like stations, parks, and schools. In addition, infectious diseases prevail in less developed areas because of the worse living environment, unstable electricity, and low income, which restrict the local residents from receiving proper clinical diagnoses. However, conventional PCR devices are usually used in indoor labs. Due to the limitations of large size, high power condition, and complicated operation, they are not suitable for clinical diagnoses in outdoor environments. Portable PCR devices can perform rapid diagnoses well in these areas. It is necessary to develop a portable PCR device for outdoor clinical diagnoses, which has a small size and features battery-powered operation and user-friendly control.

Conventional PCR devices are generally made up of three parts: a thermal cycler, reaction chambers, and a detection system. The thermal cycler usually consists of a heating component, a cooling component, and a thermal signal controller. Peltier heaters are commonly used as the heating component [12,13,14]. They usually need a high-power supply and exhibit poor thermal dissipation, thereby requiring an external fan for cooling. Polypropylene (PP) and polycarbonate (PC) are commonly used to design the tubes for the reaction chambers, which require a bulky and complicated design of the thermal cycler to achieve temperature conditions with good thermal uniformity. In addition, PC [15], cyclic olefin polymer (COP) [16], cyclic olefin copolymer (COC) [17], polymethyl methacrylate (PMMA) [18], polyethylene terephthalate (PET) [19], and polydimethylsiloxane (PDMS) [20] are frequently used to fabricate PCR chips; however, these most commonly used materials have many limitations, such as poor thermal conductivity, water permeability, and air permeability. The common methods for detecting the PCR product are agarose electrophoresis detection and fluorescence microscopy detection, both of which inevitably require a high power supply and complicated operation.

In recent years, portable equipment devices have received considerable attention in almost every field for fast and on-site operations [21,22,23]. In particular, portable PCR devices have drawn attention from various research groups. Herold et al. developed a thermocycler for PCR amplification based on thin-film resistor to amplify genes from three bacterial pathogens. The PCR results were detected using gel electrophoresis [24]. Jeong et al. developed a portable low-power thermal cycler based on Pt thin-film heater to amplify *Escherichia coli* genomic DNA and the PCR results were also detected using gel electrophoresis. Both of these methods lacked a miniaturized detection system, which blocks their utilization in portable applications [25]. Mendoza-Gallegos et al. developed a portable thermocycler based on power resistor and amplified 18S rRNA housekeeping gene and real-time detection. The heat block presented a poor performance on heating and cooling rate which restricted the system speed [26].

Polycrystalline or metallic materials are deposited on the surface of the substrate to form thin-film heaters [27,28]. Thin-film heaters enable low power consumption and fast operation due to the small thermal mass. Metallic materials are commonly used for resistance temperature detectors (RTDs); the temperature monitoring relies on the relationship between the resistance and the temperature [29,30,31]. Thin-film heaters and RTDs have been widely applied in sensor chips, biological chips, and microfluid chips [32,33]. Platinum and gold are commonly used to enable a wide range of temperature control with high stability and efficiency for thin-film heaters and RTDs. Here, we substituted platinum and gold with aluminum as the material of thin-film heaters and RTDs since aluminum has excellent thermoelectric properties and an acceptable linear proportion between temperature and the resistance in the range from 0 to 120 °C. This temperature range is enough for PCR reactions. In addition, aluminum is more easily used to perform thin-film metallization on the substrate and is much cheaper than platinum or gold.

In this study, we developed a portable PCR device which integrated a PCR thermal control system, a PCR reaction chip, and the end-point fluorescence detection system. The PCR thermal control system was developed using aluminum thin-film heaters and RTDs to provide fast and precise temperature conditions under the control of external drive circuits for outdoor applications. The disposable PCR reaction chips were made up of silicon substrate, silicone rubber, and quartz plate, which formed two chambers for the experimental group and the control group, respectively. The miniaturized fluorescence detection system consisted of a CMOS camera, an LED excitation light source and mirror units. We used *HPV16E6* genomic DNA for the PCR experiment under appropriate conditions and analyzed the results after the last cycle.

## 2. Materials and Methods

### 2.1. PCR Thermal Control System

#### 2.1.1. PCR Thermal Control Chip

The PCR thermal control system was made up of a PCR thermal control chip and external drive circuits. Based on the principle of the Joule effect, the thin-film heaters generated heat when a voltage was applied on both sides of the thin-film resistance. As for the RTDs, the resistance was defined by the resistivity of the thin-film material and the length and cross-sectional area of the thin-film resistance. There was a linear relationship between resistivity and temperature when the temperature did not change much. The thin-film heater and the RTDs can be fabricated on a variety of substrates such as silicon, glass, and photopolymer depending on the different requirements. Different materials can provide distinct superior properties to fit the application environment. Here, we took silicon as the substrate because of its excellent thermal conductivity, which makes the system more efficient. In addition, silicon wafers are most commonly used in large-scale semiconductor manufacturing, which can greatly reduce the fabricating costs.

**Figure 1:**
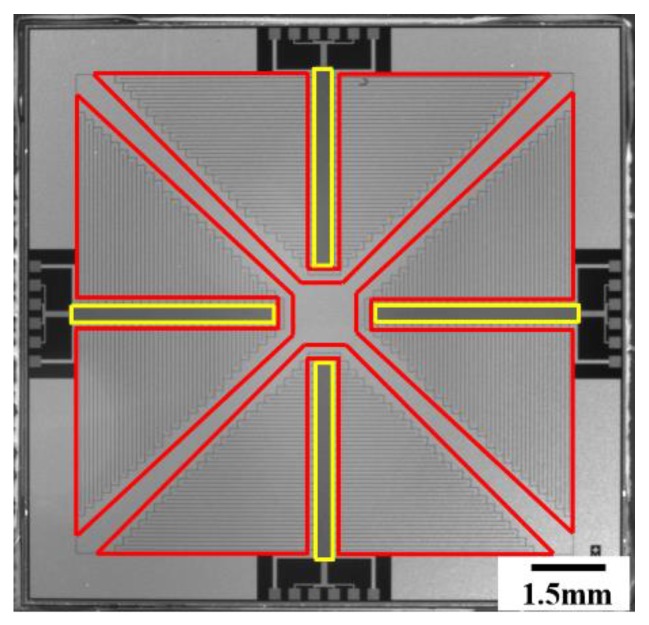
Photograph of the thin-film heaters and resistance temperature detectors (RTDs) on the polymerase chain reaction (PCR) thermal control chip. The areas delineated by the red lines are the thin-film heaters and the areas delineated by the yellow lines are the RTDs.

**Figure 2:**
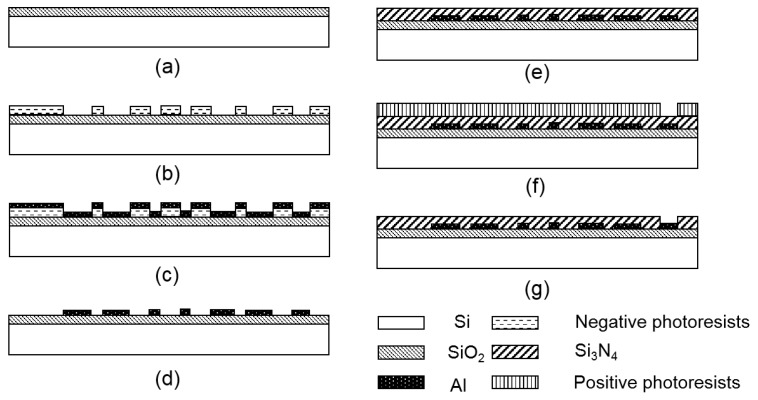
The fabricating processes of the thin-film heaters and RTDs. (**a**) Thermal oxidation for SiO_2_ layer. (**b**) Lithography development for aluminum deposition. (**c**) Aluminum deposition by electron beam evaporation. (**d**) Lift-off. (**e**) Si_3_N_4_ deposition by PECVD. (**f**) Lithography development for etching windows. (**g**) Etching for aluminum pads.

The chip contained four thin-film heaters and four RTDs, and the patterns of the thin-film heaters and RTDs are shown in Figure 1. Four serpentine thin-film heaters surrounded four RTDs to provide excellent thermal uniformity and accurate monitoring. The thin-film heaters and RTDs were fabricated on a silicon wafer by using conventional semiconductor technology as illustrated in Figure 2. The pre-cleaned wafer was thermally oxidated to form a dielectric layer as shown in Figure 2a; the thickness of the SiO_2_ layer was 500 nm. Negative photoresists were spin-coated on the wafer and developed to form the patterns of the thin-film heaters and RTDs as shown in Figure 2b. Due to the stable adhesion characteristics between aluminum and SiO_2_, aluminum was directly deposited on the surface of the photoresists and the substrate to form the thin-film heaters and RTDs by using electron beam evaporation, as shown in Figure 2c. The photoresists were removed, and the thin-film heaters and RTDs were left on the surface of the substrate after immersing the wafer in acetone for 24 h, as shown in Figure 2d. Si_3_N_4_ was deposited on the top to form a passivation layer by using plasma-enhanced chemical vapor deposition (PECVD), as shown in Figure 2e; the thickness of the Si_3_N_4_ layer was 2 μm. Positive photoresists were spin-coated on the wafer and developed to open the etching window for aluminum pads, as shown in Figure 2f. Finally, the exposed Si_3_N_4_ was etched to expose the aluminum pads, and the positive photoresists were removed, as shown in Figure 2g. The PCR thermal control chip was 15 mm × 15 mm. All the thin-film heaters and RTDs were fabricated on the silicon substrate using mature semiconductor technologies, which ensured the uniformity and repeatability of the PCR thermal control chip.

#### 2.1.2. External Drive Circuits

**Figure 3:**
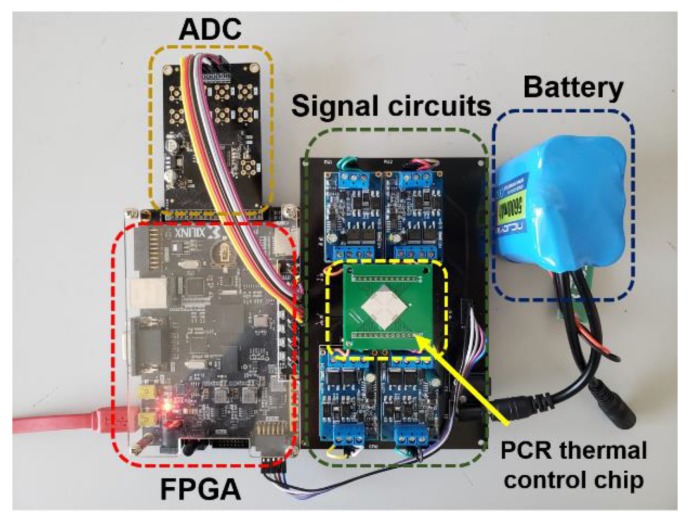
Photograph of the PCR thermal control chip with the external driver circuits.

**Figure 4:**
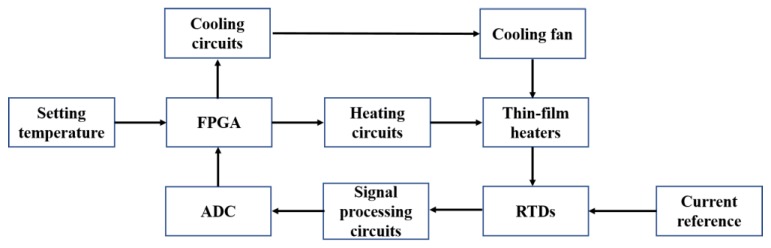
Block diagram of the control processes.

In addition to the thin-film heaters and RTDs, external drive circuits were also important components of the PCR thermal control system. The PCR thermal control chip was packaged on a PCB and connected to the external drive circuits as shown in Figure 3. The block diagram in Figure 4 shows the control processes. The heaters were controlled using proportional-integral-derivative (PID) closed-loop controllers, which were implemented on a field-programmable gate array (FPGA) (AX309, ALINX) and driven by battery-powered driving circuits. The RTDs were driven by rectifying circuits and transmitted the real-time temperature signals to the FPGA through signal processing and sampling circuits. The cooling procedure was implemented by a mini fan (AD01703HX04AB00, ADDA Corp., Ltd.) based on the demands. The cooling fan is shown in Supplementary Figure 1.

**Figure 5:**
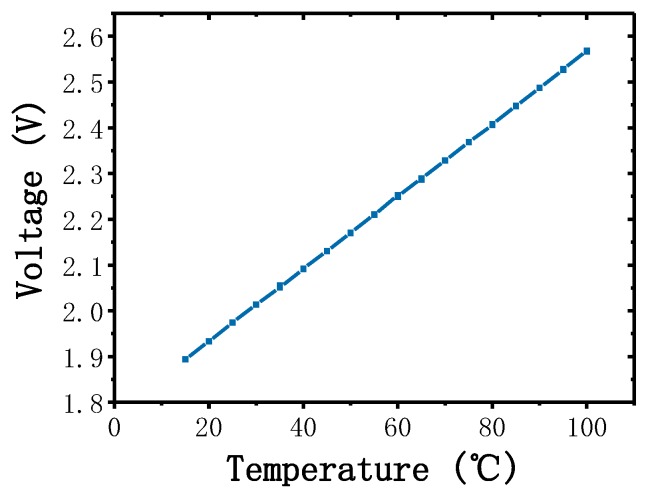
Linear functional relationship between the temperature and voltage of the thin-film heater.

All the outer control components were powered by a 24 V Li-ion battery and the capacity of the battery was 6000 mAH. The operating voltages of the components were different; hence, they were optimized at 5 V for the FPGA and cooling fan and at 15 V for the rest of the components. LM2577 is a simple voltage regulator that can step-up or step-down the input voltage to the required voltage. Two LM2577 regulators were used here to regulate the battery voltage at 5 and 15 V. The thin-film heaters were driven using electronic switches based on a high-power MOSFET AOD2810, which provided a high current output and low inter-resistance. We developed a PID controller on the FPGA. The PID controller provided pulse-width modulation (PWM) signals to the electronic switches based on the real-time temperature feedback. The electronic switches opened when the PWM signals were at a high level and closed when the PWM signals were at a low level depending on the duty cycles of the PWM signals. The fan was also enabled by an electronic switch, which was controlled by the FPGA, the fan worked only when there were cooling demands. The RTDs gave the temperature signals of the thin-film heaters, depending on the material properties, the resistances of the RTDs varied with changes in temperature. To achieve precise temperature control, we used 4-wire measurements to eliminate contact resistances and lead resistances. A precision voltage-to-current convertor XTR111 was used here to produce an accurate current reference for the RTDs, and a low-power high-accuracy instrumentation amplifier AD620 was used to amplify the resistance of the RTDs. A low-power dual-operational amplifier LM358 was used as the voltage follower. The voltage–temperature relationship of the RTD was measured and is shown in Figure 5. An 8-channel 16-bit analog-to-digital converter (ADC) AD7606 was used to convert and transmit the temperature signals to the FPGA; the ADC was powered and driven by the FPGA. The average power consumption of the PCR thermal control system was approximately 6.5 W and the battery could power the system for at least 15 h with full consideration of power loss.

#### 2.1.3. PCR Reaction Chip

**Figure 6:**
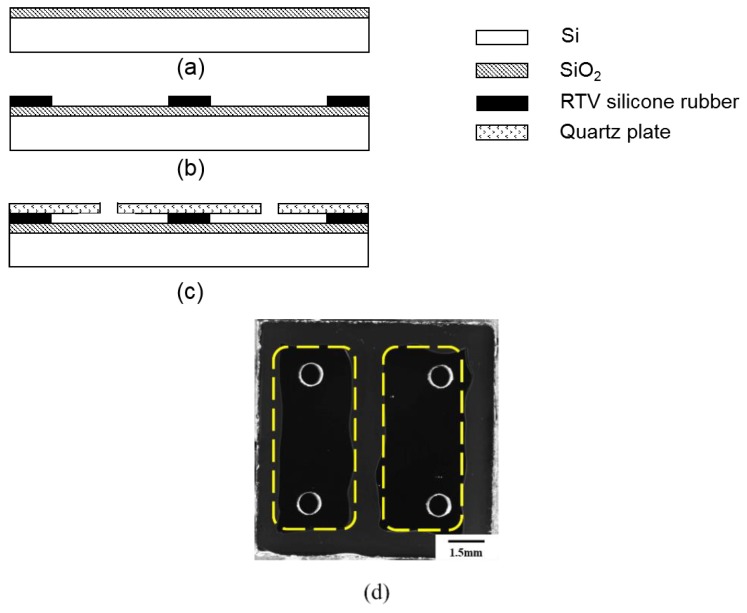
The fabricating processes and the planform of the PCR reaction chamber. (**a**) Thermal oxidation for SiO_2_ layer. (**b**) Patterning of the RTV silicone rubber. (**c**) Quartz plate attaching. (**d**) Two chambers on a reaction chip (circled by yellow dotted line); each chamber had an inlet and an outlet. The volume of each chamber was 10 μL.

A sealed chamber with good conduction of heat and biological compatibility is required to provide a reaction place for PCR. The PCR reaction chip was made up of a silicon substrate, room temperature vulcanized (RTV) silicone rubber, and quartz plate. We attached the disposable reaction chip to the PCR thermal control chip using thermally conductive silicone grease. The silicon substrate had excellent thermal conductivity, which provided good thermal uniformity for the PCR reaction. The RTV silicone rubber, which exhibited good stability in the temperature range of −60~200 °C, had preferable adhesion to bond the silicon substrate and the quartz plate together. The visible light transmittance of the quartz plate is more than 90%, which makes the quartz plate suitable for transmitting a fluorescence signal through it. In addition, all the materials used here were suitable for PCR reactions, as they were gas-proof, water-proof, and biocompatible. Figure 6 illustrates the detailed fabricating processes and planform of the PCR reaction chip. First, the silicon substrate was thermally oxidated to produce a SiO_2_ layer, as shown in Figure 6a; the thickness of the SiO_2_ layer was 200 nm. Then, the RTV silicone rubber was patterned on the surface of the substrate to shape the reaction chamber, as shown in Figure 6b; the thickness of the RTV silicone rubber was 200 μm. The quartz plate, which had been drilled for inlets and outlets beforehand by using a computerized numerical control machine, was attached to the RTV silicone rubber layer as the upper cover plate as shown in Figure 6c; the thickness of the quartz plate was 1 mm. Finally, the curing condition of RTV silicone rubber was 24 h in room temperature environment. The planform of the PCR reaction chip is shown in Figure 6d. Silicone rubber plugs were used for sealing the PCR reaction chip after injecting the PCR reagents. The volume of a single chamber was 10 μL, and the size of the PCR reaction chip was 11 mm × 11 mm. Compared to conventional PCR tubes and chips, the thin-film reaction chambers shaped the PCR reagents into approximately two-dimensional surfaces with a minuscule thickness of 200 μm. The thin reaction chamber provided better efficiency of heat utilization and reduced the consumption of PCR reagents. In addition, the fabricating processes of the disposable reaction chip were simple and the materials were easy to prepare and were cheap, which could sufficiently reduce the cost of the disposable chips. The cost was expected to be less than USD 0.50 after entering the stage of large-scale industrial production.

### 2.2. Fluorescence Detection System

**Figure 7:**
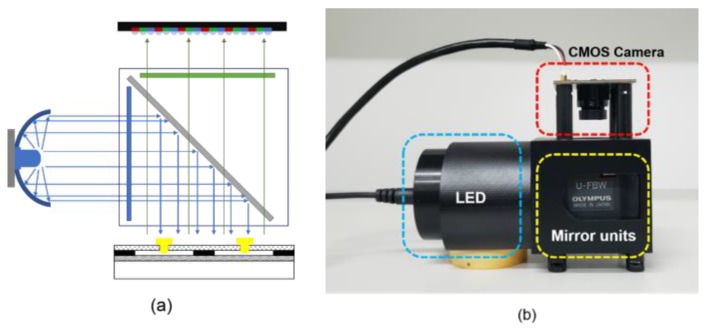
Fluorescence detection system. (**a**) Schematic diagram of the detection optical path. (**b**) Photograph of the 3D-printed structure with the integrated components.

A fluorescence detection system was designed to detect the PCR results. The detection system was made up of a CMOS camera (OV2710, OmniVision, Santa Clara, CA, USA), a blue LED (XLamp XP-E, Cree, Inc., Durham, NC, USA), and mirror units (U-FBW, Olympus, Tokyo, Japan). The results were displayed on a laptop. The mirror units contained a dichroic mirror, an excitation filter, and an emission filter. The blue LED was placed on the side of the filters and combined with a circular reflector to reduce the light decay. The reaction chips were illuminated by the blue LED, and the dominant wavelength of the LED was 465~485 nm. The CMOS camera captured the fluorescence signal through the emission filter as shown in the schematic diagram in Figure 7a. A 3D-printed structure integrated the LED, the CMOS camera, and the mirror units together as shown in Figure 7b. The blue LED, which had a power consumption of 3 W, was powered by the Li-ion battery. The CMOS camera was powered and driven by the laptop.

### 2.3. PCR Reagents

To evaluate the performance of our portable PCR device, we amplified *HPV16E6* genomic DNA and the DNA template was 200 copies/μL. The reaction reagents included PCR mix (Code No.QPS-101, Toyobo Co., Ltd., Osaka, Japan), forward primer (Sangon Biotech Shanghai Co., Ltd., Shanghai, China), reverse primer (Sangon Biotech Shanghai Co., Ltd., Shanghai, China), probe (Sangon Biotech Shanghai Co., Ltd., Shanghai, China) and nuclease-free water (9012, TaKaRa, Berkeley, CA, USA). The probe sequence was AGGAGCGACCCGGAAAGTTACCACAGTT, the forward primer sequence was CTGCAATGTTTCAGGACCCA and the reverse primer sequence was TCATGTATAGTTGTTTGCAGCTCTGT. Table 1 shows the volume of these reagents required to prepare 10 μL PCR reagents (experimental group and control group).

## 3. Results and Discussion

### 3.1. Temperature Control Performance

**Figure 8:**
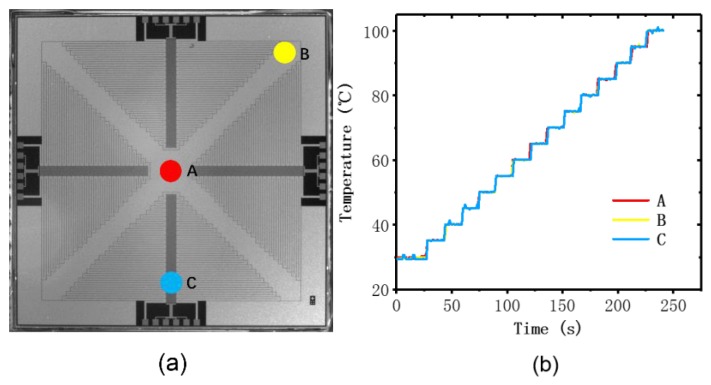
Heating uniformity experiment by monitoring three places on the PCR thermal control chip. (**a**) The positions at which the temperature was monitored: in the middle (red, A) and at the edges (yellow and blue, B and C). (**b**) The temperature traces of the three monitoring points measured by PT100.

To evaluate the performance of the thin-film heater and the RTDs, we carried out several experiments to verify the thermal uniformity, accuracy, and precision of the temperature measurement. Temperature uniformity is an essential condition for successful PCR reaction, since a heterogeneous heat distribution may cause the failure of the PCR reaction. A commercial Pt100 was placed at different places on the surface of the PCR thermal control chip to measure the uniformity. We attached the Pt100 to the chip using thermally conductive silicone grease and took measurements at three monitoring points on the surface (A–C) as shown in Figure 8a. We set the start point of the test at 35 °C and the last point of the test was 100 °C. At every 5 °C, we set a test point, and the dwell time at each test point was 15 s. The temperature differences between these points were found to be less than 0.76 °C, as shown in Figure 8b. The heat distribution over the surface of the PCR thermal control chip exhibited good uniformity at different temperature setting in the range of 35 to 100 °C, and we give the credit for this to the proper routing design and excellent thermal conductivity of the silicon substrate.

**Figure 9:**
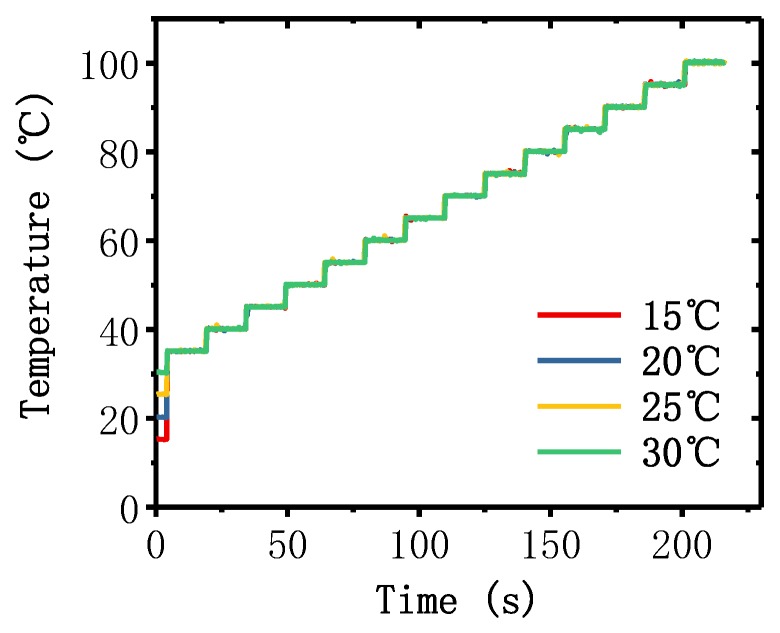
Temperature traces of the PCR thermal control chip under different environmental temperatures (15, 20, 25, and 30 °C) measured by RTDs. The heating rate in the lower temperature range was much higher than the average heating rate, and the time consumption of heating to the start point (35 °C) was negligible; hence, the temperature traces from the environmental temperature to the start point (35 °C) were approximately coincident.

For the PCR reaction, accurate and stable temperature control at a specific temperature setting is indispensable. Several experiments were conducted to verify the stability and precision of the PCR thermal control system under different environments. We set the start point of the test at 35 °C and the last point of the test at 100 °C. For every 5 °C, we set a test point, and the dwell time at each test point was 15 s. We repeated the test with different environmental temperatures: 15, 20, 25, and 30 °C. The temperature measured by using RTDs are shown in Figure 9. It can be seen that the temperature increased quickly to respond to the variation in the setting temperature, and the overshoot was insignificant. Moreover, the precise temperature control in the different environment showed good performance in the repeatability of the PCR thermal control system. We evaluated the accurate temperature control of the chip by defining the accuracy as the difference between the temperature of the test points and the average temperature measured by the RTDs. In addition, we defined the average accuracy as the average value of the accuracies at all test points. The average accuracies in the temperature range, 35~10 °C, were 0.144, 0.145, 0.151, and 0.145 °C when the environmental temperature was 15, 20, 25, and 30 °C, respectively. The average heating rate was 32 °C/s and the average cooling rate was 7.5 °C/s, which was much faster than other portable and battery-powered PCR devices as shown in Table 2. The detailed results of the average heating/cooling rate are discussed in Supplementary Note 1.

### 3.2. Reaction Chip Evaluation and Optical Setup

**Figure 10:**
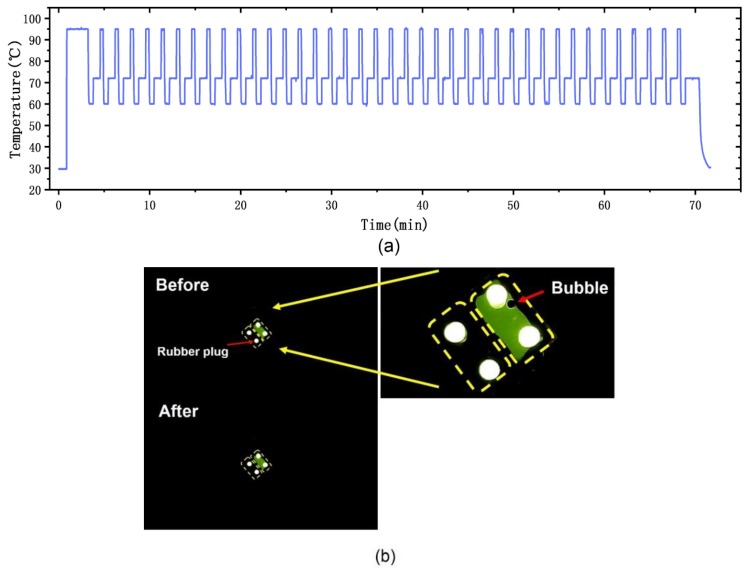
Performance test for the PCR reaction chip and the fluorescence detection system. (**a**) The temperature trace of the PCR protocol. (**b**) PCR reaction chip before and after conventional PCR protocol. Bubbles were sometimes generated when the reagents were injected to the reaction chip using a pipette. The enlarged picture of the PCR reaction chip showed the generated bubble.

We evaluated the sealing performance, precision of the temperature, and the performance of the optical setup. We used fluorescein to verify the reliability of the PCR reaction chip and fluorescence detection system; the fluorescein reagent was injected into the top-right chamber of the PCR reaction chip and deionized water was injected into the bottom-left chamber using a pipette. The reaction chip was sealed by silicone rubber plugs. To simulate the PCR environment, we implemented a conventional PCR protocol that included a pretreatment at 95 °C for 2 min for initial denaturation, followed by 40 PCR cycles, which consisted of three procedures (denaturation: 95 °C for 20 s, annealing: 60 °C for 30 s, and elongation: 72 °C for 45 s). The environmental temperature was 28.5 °C. Figure 10a shows the temperature trace of this protocol, where the temperature was measured by the RTDs on the PCR thermal control chip. We evaluated the precision of the chip by defining it as the average of the standard deviations of the test points. The precision of the chip was 0.182, 0.179, and 0.087 °C at 95, 60, and 72 °C, respectively. The results were captured by the CMOS camera and transmitted to the laptop. A striking contrast was observed by the detection system. Figure 10b shows the PCR reaction chip before and after the PCR protocol; there was no reagent leakage or crosstalk between the two chambers under cyclic high-temperature heating.

### 3.3. PCR Experiments and Detection

**Figure 11:**
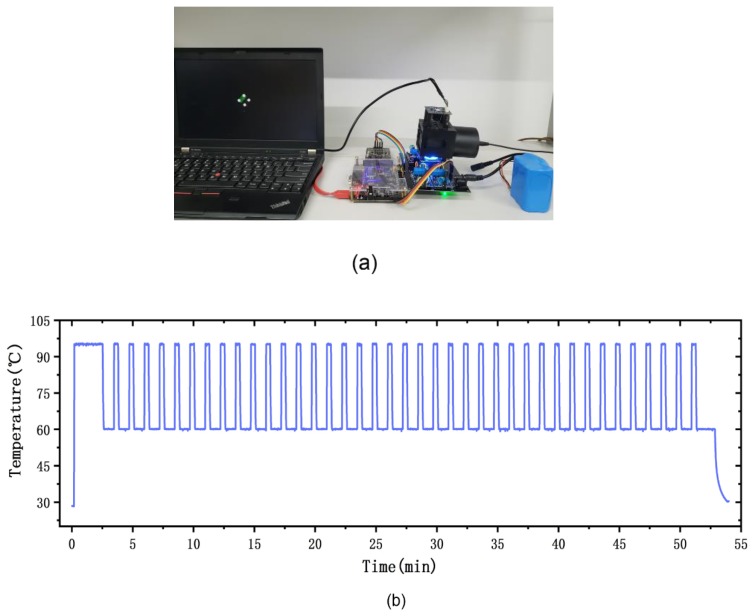

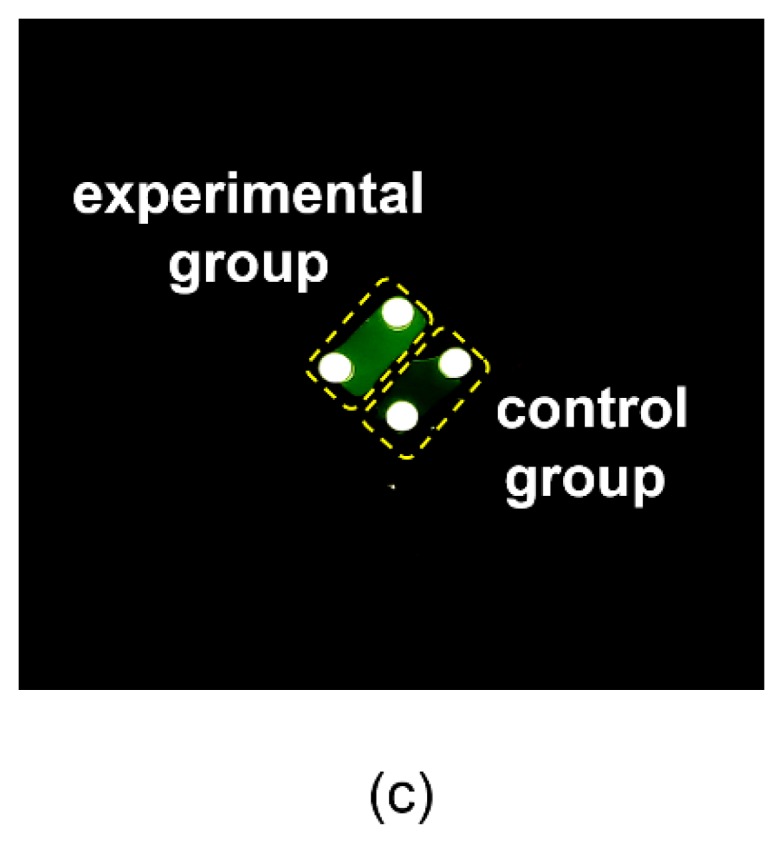
PCR amplification experiments with *HPV16E6* genomic DNA. (**a**) Photograph of the portable PCR device and the experimental environment. (**b**) The temperature trace of the 40-cycle PCR condition for *HPV16E6* genomic DNA. (**c**) The results obtained by our fluorescence detection system. There was significant fluorescence difference between the experimental group and the control group.

To evaluate the performance of our portable PCR device, we amplified *HPV16E6* genomic DNA using our device as shown in Figure 11a. The experimental conditions used for PCR amplification of *HPV16E6* genomic DNA were 95 °C for 2 min (initialization), 95 °C for 20 s (denaturation), 60 °C for 50 s (annealing and elongation), and we implemented 40 cycles. The environmental temperature was 28 °C. The top-left chamber contained the experimental group and the bottom-right chamber contained the control group. The PCR reaction chip was sealed by silicone rubber plugs. The temperature trace of the thermal control chip is shown in Figure 11b. After the PCR protocol, the end-point detection result was captured by the fluorescence detection system, as shown in Figure 11c. To support our results, we also amplified *HPV16E6* genomic DNA using a commercial lab thermal cycler (Applied Biosystems 2720 Thermal 294 Cycler, Thermo Fisher Scientific, Waltham, MA, USA). The detailed results of the lab thermal cycler are discussed in Supplementary Note 2. The time consumption of the entire PCR amplification was below 1 h. The PCR speed was determined by the heating/cooling time consumption and the time consumption of the three PCR procedures (denaturation, annealing and elongation), our fast heating/cooling rate reduced the heating/cooling time consumption to 4 min.

**Figure 12:**
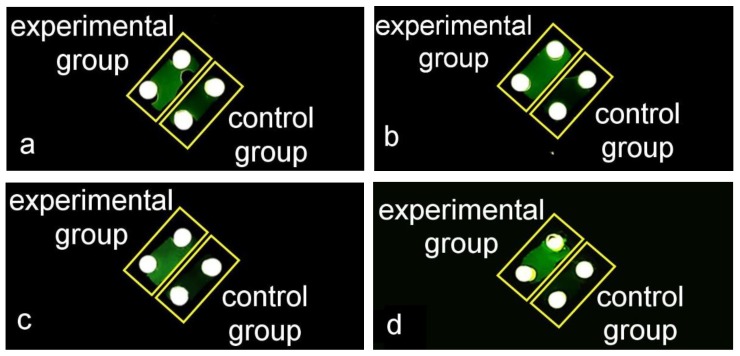
The results of amplifying HPV16E6 genomic DNA samples in different initial concentrations. The top-left chambers were the experimental groups and the bottom-right chambers were the control groups. (**a**) The results of amplifying DNA sample 1. (**b**) The results of amplifying DNA sample 2. (**c**) The results of amplifying DNA sample 3. (**d**) The results of amplifying DNA sample 4.

DNA samples in different initial concentrations were amplified and detected using our portable device. The concentrations of the DNA samples are shown in Table 3. The PCR reaction reagents were prepared according to Table 1 and the experiment conditions were in accord with the conditions we used before (initialization: 95 °C for 2 min, denaturation: 95 °C for 20 s, annealing and elongation: 60 °C for 50 s, and we implemented 40 cycles). The results of the amplifications of DNA samples in different concentrations are shown in Figure 12. There were significant fluorescence differences between the experimental groups and the control groups, which indicated that the portable device was able to amplify and detect DNA samples in different initial concentrations.

## 4. Conclusions

We developed a portable PCR device to meet the requirements of outdoor nucleic acid amplification and detection, which was made up of three modules: PCR thermal control system, PCR reaction chip, and fluorescence detection system. The PCR thermal control system consists of a thermal control chip (aluminum thin-film heaters and RTDs) and external circuits, which provide fast and stable temperature control. Aluminum heaters were optimally designed with serpentine lines to achieve uniformity of thermal distribution, and the aluminum RTDs were surrounded by the thin-film heaters to achieve precise temperature monitoring. A 24 V Li-ion battery powered the thermal control chip and external circuits. The PCR reaction chip was made up of a silicon substrate, RTV silicone rubber, and a quartz plate. The reaction chip was disposable, and the preparation process was simple and convenient. The fluorescence detection system was made up of a CMOS camera, an excitation light source, and mirror units. The portable CMOS camera was driven and powered by a laptop, and the end-point results were captured and displayed on the laptop. The excitation light source was also powered by the Li-ion battery.

The device achieved fast and precise temperature regulation with an average heating rate of 32 °C/s and an average cooling rate of 7.5 °C/s. This device allowed us to perform nucleic acid amplification and end-point detection under different environmental conditions. *HPV16E6* genomic DNA was used to verify the performance of the device. The reliable detection results prove the practicability and good performance of our portable PCR device. Our portable PCR device provides a useful and reliable tool for outdoor PCR amplification and on-site testing. In further research, we will optimize the display module. We will consider replacing the laptop with a tablet. Moreover, real-time PCR will be the next goal for our portable PCR device, which could provide quantitative analysis for DNA detection.

## Figures and Tables

**Table 1:** Ratio of reaction reagents for PCR amplification.

Reagents	Copy Template	Nuclease-Free Water	Forward Primer	Reverse Primer	Probe	PCR Mix	Total Reagents
**Experimental group (μL)**	0.5	3.7	0.3	0.3	0.2	5	10
**Control group (μL)**	0	4.2	0.3	0.3	0.2	5	10

**Table 2:** Comparison of average heating rate and cooling rate between our work and other portable and battery-powered PCR devices.

Devices	[15]	[17]	[18]	Our Work
**Average heating rate**	6–7 °C/s	1.08 °C/s	0.5 °C/s	32 °C/s
**Average cooling rate**	5 °C/s	0.9 °C/s	1.4 °C/s	7.5 °C/s

**Table 3:** Different initial concentrations of the DNA samples.

DNA Samples	1	2	3	4
**Concentration (*copies*/μL)**	150	200	500	1000

## Supplementary Materials

The following are available online at https://www.mdpi.com/1424-8220/20/9/2627/s1. Figure S1. Photograph of the cooling fan; Figure S2. Heating/cooling curves. (a) the heating cure of the PCR thermal control chip from 28 °C to 95 °C. (b) the cooling cures of the PCR thermal control chip from 95 °C to 60 °C, the working condition of the fan affected the cooling rate; Figure S3. The results of HPV16E6 genomic DNA amplification using the thermal cycler and fluorescence detection microscope; Table S1. Ratio of reaction reagents for PCR amplification.

## References

[1] Mullis K., Faloona F., Scharf S., Saiki R., Horn G., Erlich H. (1986). Specific enzymatic amplification of DNA in vitro: The polymerase chain reaction. Cold Spring Harb. Symp. Quant. Biol..

[2] Saiki R.K., Gelfand D.H., Stoffel S., Scharf S.J., Higuchi R., Horn G.T., Mullis K., Erlich H.A. (1988). Primer-directed enzymatic amplification of DNA with a thermostable DNA polymerase. Science.

[3] Mullis K. (1990). The unusual origin of the polymerase chain reaction. Sci. Am..

[4] Kenneth J.L., Thomas D.S. (2001). Analysis of relative gene expression data using real-time quantitative PCR and the 2^−∆∆*C_T_*^ Method. Methods.

[5] Van Guilder H.D., Vrana K.E., Freeman W.M. (2008). Twenty-five years of quantitative PCR for gene expression analysis. Biotechniques.

[6] Schönian G., Nasereddin A., Dinse N., Schweynoch C., Schallig H.D.F.H., Presber W., Jaffe C.L. (2003). PCR diagnosis and characterization of Leishmania in local and imported clinical samples. Diagn. Microbiol. Infect. Dis..

[7] Bustin S.A., Mueller R. (2005). Real-time reverse transcription PCR (qRT-PCR) and its potential use in clinical diagnosisb. Clin. Sci..

[8] Piknová L., Pangallo D., Kuchta T. (2008). A novel real-time polymerase chain reaction (PCR) method for the detection of hazelnuts in food. Eur. Food Res. Technol..

[9] Cocolin L., Rajkovic A., Rantsiou K., Uyttendaele M. (2011). The challenge of merging food safety diagnostic needs with quantitative PCR platforms. Trends Food Sci. Technol..

[10] Nakanishi H., Kido A., Ohmori T., Takada A., Hara M., Adachi N., Saito K. (2009). A novel method for the identification of saliva by detecting oral streptococci using PCR. Forensic Sci. Int..

[11] Power D.A., Cordiner S.J., Kieser J.A., Tompkins G.R., Horswell J. (2010). PCR-based detection of salivary bacteria as a marker of expirated blood. Sci. Justice.

[12] Wittbrodt J., Erhardt W. (1989). An inexpensive and versatile computer-controlled PCR machine using a Peltier element as thermoelectric heat pump. Trends Genet..

[13] Khandurina J., McKnight T.E., Jacobson S.C., Waters L.C., Foote R.S., Ramsey J.M. (2000). Integrated system for rapid PCR-based DNA analysis in microfluidic devices. Anal. Chem..

[14] DuVall J.A., Roux D.L., Tsuei A.C., Thompson B.L., Birch C., Li J., Nelson D.A., Mills D.L., Ewing M.M., McLaren R.S. (2016). A rotationally-driven polyethylene terephthalate microdevice with integrated reagent mixing for multiplexed PCR amplification of DNA. Anal. Methods.

[15] Haber J.M., Gascoyne P.R.C., Sokolov K. (2017). Rapid real-time recirculating PCR using localized surface plasmon resonance (LSPR) and piezo-electric pumping. Lab. Chip..

[16] Czilwik G., Messinger T., Strohmeier O., Wadle S., von Stetten F., Paust N., Roth G., Zengerle R., Saarinen P., Niittymäki J. (2015). Rapid and fully automated bacterial pathogen detection on a centrifugal-microfluidic LabDisk using highly sensitive nested PCR with integrated sample preparation. Lab. Chip..

[17] Lee S.H., Kim S., Kang J.Y., Ahna C.H. (2008). A polymer lab-on-a-chip for reverse transcription (RT)-PCR based point-of-care clinical diagnostics. Lab. Chip..

[18] Gan W., Gu Y., Han J., Li C., Sun J., Liu P. (2017). Chitosan-Modified Filter Paper for Nucleic Acid Extraction and “*in Situ* PCR” on a Thermoplastic Microchip. Anal. Chem..

[19] Bae N.H., Lim S.Y., Song Y., Jeong S.W., Shin S.Y., Kim Y.T., Lee T.J., Lee K.G., Lee S.J., Oh Y.J. (2018). A Disposable and Multi-Chamber Film-Based PCR Chip for Detection of Foodborne Pathogen. Sensors.

[20] Lee D., Kim Y.T., Lee J.W., Kim D.H., Seo T.S. (2016). An integrated direct loop-mediated isothermal amplification microdevice incorporated with an immunochromatographic strip for bacteria detection in human whole blood and milk without a sample preparation step. Biosens. Bioelectron..

[21] Kim J., Byun D., Mauk M.G., Bau H.H. (2009). A disposable, self-contained PCR chip. Lab. Chip..

[22] Mahato K., Srivastava A., Chandra P. (2017). Paper based diagnostics for personalized health care: Emerging technologies and commercial aspects. Biosens. Bioelectron..

[23] Syedmoradi L., Daneshpour M., Alvandipour M., Gomez F.A., Hajghassem H., Omidfar K. (2017). Point of care testing: The impact of nanotechnology. Biosens. Bioelectron..

[24] Herold K.E., Sergeev N., Matviyenko A., Rasooly A. (2009). Rapid DNA amplification using a battery-powered thin-film resistive thermocycler. Methods Mol. Biol..

[25] Jeong S., Lim J., Kim M.Y., Yeom J., Cho H., Lee H., Shin Y.B., Lee J.H. (2018). Portable low-power thermal cycler with dual thin-film Pt heaters for a polymeric PCR chip. Biomed. Microdevices.

[26] Mendoza-Gallegos R.A., Rios A., Garcia-Cordero J.L. (2018). An Affordable and Portable Thermocycler for Real-Time PCR Made of 3D-Printed Parts and Off-the-Shelf Electronics. Anal. Chem..

[27] Dai C.L. (2007). A capacitive humidity sensor integrated with micro heater and ring oscillator circuit fabricated by CMOS–MEMS technique. Sens. Actuators B Chemical.

[28] Resnik D., Vrtačnik D., Aljančič U., Možek M., Amon S. Experimental study of Ti/Pt thin film heater and temperature sensors on Si platform. Proceedings of the SENSORS, 2009 IEEE.

[29] Yan W., Li H., Liu J., Guo J. (2007). EPMA and XRD study on nickel metal thin film for temperature sensor. Sens. Actuators A Phys..

[30] Yi C., Lee J.H., Kwak B.S., Lin M.X., Kim H.O., Jung H.I. (2014). Diagnosis of diabetes mellitus using sialic acid expression of erythrocyte and a microfluidic resistive temperature detector (micro-RTD). Sens. Actuators B Chem..

[31] Han J., Cheng P., Wang H., Zhang C., Zhang J., Wang Y., Duan L., Ding G. (2014). MEMS-based Pt film temperature sensor on an alumina substrate. Mater. Lett..

[32] Johnson C.L., Wise K.D., Schwank J.W. A Thin-Film Gas Detector for Semiconductor Process Gases. Proceedings of the Technical Digest., International Electron Devices Meeting.

[33] Zhao Z., Cui Z., Cui D., Xia S. (2003). Monolithically integrated PCR biochip for DNA amplification. Sens. Actuators A Phys..

