# Supplementary material for: Portable and Battery-Powered PCR Device for DNA Amplification and Fluorescence Detection
Source: Sensors (Basel). 2020 May 5;20(9):2627. doi: 10.3390/s20092627 (PMC7249063; doi:10.3390/s20092627)
Supplement: Supplementary file 1 [file sensors-20-02627-s001.pdf]

sensors

Supplementary Materials

Portable and Battery-Powered PCR Device for DNA Amplification and Fluorescence Detection

Junyao Jie [1,2], Shiming Hu [1,2], Wenwen Liu [1], Qingquan Wei [1], Yizheng Huang [1,2], Xinxin Yuan [1,2], Lufeng Ren [1], Manqing Tan [1,2] and Yude Yu [1,2,*]

1 State Key Laboratory of Integrated Optoelectronics, Institute of Semiconductors, Chinese Academy of Sciences, P.O. Box 912, Beijing 100083, China; jiejunyao@semi.ac.cn (J.J.); hushiming@semi.ac.cn (S.H.); liuwenwen@semi.ac.cn (W.L.); qingquanwei@semi.ac.cn (Q.W.); yzhuang@red.semi.ac.cn (Y.H.); yuanxinxin@semi.ac.cn (X.Y.); renlf@semi.ac.cn (L.R.); mqtan@semi.ac.cn (M.T.)

2 College of Materials Science and Opto-Electronic Technology, University of Chinese Academy of Sciences

* Correspondence: yudeyu@semi.ac.cn; Tel.: +86-10-823-049-79

The PCR thermal control chip was packaged on a hollowed-out PCB, and the back of the PCR thermal control chip was exposed. The cooling fan was installed on the back of the PCR thermal control chip to cool the chip according to the control signals.

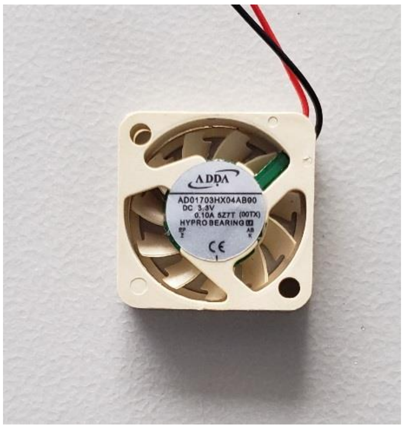

Supplementary Figure 1. Photograph of the cooling fan.

Supplementary Note 1. The PCR thermal control chip was heated by the thin-film heaters, the heaters were powered by a 24 V battery. The PCR thermal control chip was cooled by the cooling fan, and the fan was powered by a voltage regulator which provided a 5V supply. The fan was controlled by an electronic switch which received control signals from the FPGA. The average heating rate was 32 ℃/s from 28 ℃to 95 ℃ which could be calculated from **Supplementary Figure** 2 **(a)**. The average cooling rate was 7.5 ℃/s from 95 ℃to 60 ℃ when the fan was enabled and the average cooling rate was 3.2 ℃/s from 95 ℃to 60 ℃ when the fan was disabled, which could be calculated from **Supplementary Figure** 2 **(b)**. The speed of the PCR amplification was determined by the heating/cooling time consumption and the time consumption of the three PCR procedures (denaturation, annealing and elongation). Our device has reduced the heating/cooling time consumption to 4 min, the time consumption of the three PCR procedures is relevant to the DNA polymerase and the base pairs of the DNA template.

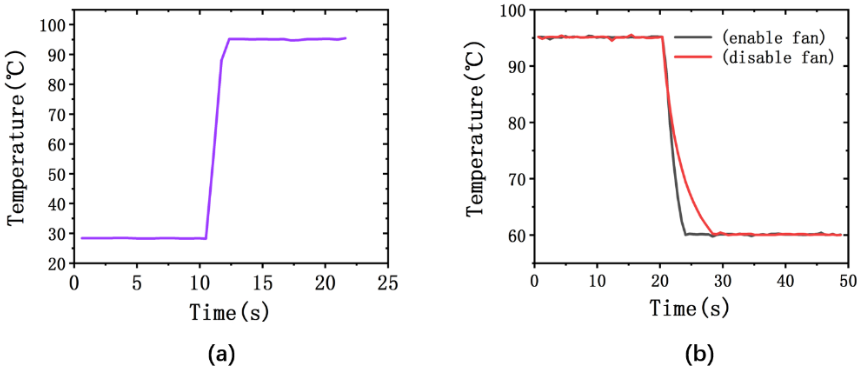

Supplementary Figure 2. Heating/cooling curves. (a) the heating cure of the PCR thermal control chip from 28 ℃to 95 ℃. (b) the cooling cures of the PCR thermal control chip from 95 ℃to 60 ℃, the working condition of the fan affected the cooling rate.

Supplementary Note 2 We used our portable device to amplify *HPV16E6* genomic DNA and detected the results by using our miniaturized fluorescence detection system. To testify the validity of our results, we used lab thermal cycler to amplify the same sample. The thermal cycler use plastic tubes to perform PCR amplification. The volume of PCR reagents we used here to perform PCR in tube was 50 μL, and the volume of the reaction reagents required to prepare 50 μL were shown in the **Supplementary Table** 1(experimental group and control group)**.** The experimental conditions used for lab thermal cycler were in accordance with the conditions used for our portable device. The max block ramp rate of the thermal cycler was 2.7 ℃/s. The amplification results were detected using a customized fluorescence microscope with a CCD (VTP23-M, LANGYAN CAMERA), as shown in **Supplementary Figure** 3. The picture was a grayscale photo, and the results were in accord with the detection results of our portable device, which can support the validity of our detection result.

Supplementary Table 1. Ratio of reaction reagents for PCR amplification.

Reagents	Copy Template	Nuclease-Free Water	Forward Primer	Reverse Primer	Probe	PCR Mix	Total Reagents
Experimental group (*μl*)	2.5	18.5	1.5	1.5	1	25	50
Control group (*μl*)	0	21	1.5	1.5	1	25	50

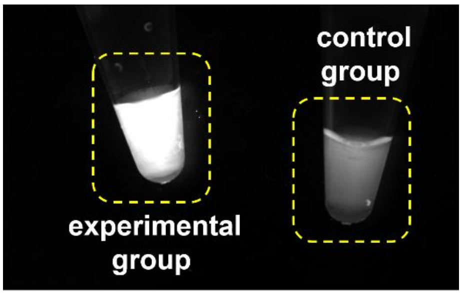

Supplementary Figure 3. The results of *HPV16E6* genomic DNA amplification using the thermal cycler and fluorescence detection microscope.

© 2018 by the authors. Submitted for possible open access publication under the terms and conditions of the Creative Commons Attribution (CC BY) license (http://creativecommons.org/licenses/by/4.0/).
